# Population Structure of Manganese-Oxidizing Bacteria in Stratified Soils and Properties of Manganese Oxide Aggregates under Manganese–Complex Medium Enrichment

**DOI:** 10.1371/journal.pone.0073778

**Published:** 2013-09-12

**Authors:** Weihong Yang, Zhen Zhang, Zhongming Zhang, Hong Chen, Jin Liu, Muhammad Ali, Fan Liu, Lin Li

**Affiliations:** 1 State Key Laboratory of Agricultural Microbiology, Huazhong Agricultural University, Wuhan, China; 2 Key Laboratory of Subtropical Agricultural Resource and Environment, Ministry of Agriculture, Huazhong Agricultural University, Wuhan, China; 3 Biotechnology Program, Department of Environmental Sciences, COMSATS Institute of Information Technology (CIIT), Abbottabad, Pakistan; University Paris South, France

## Abstract

Manganese-oxidizing bacteria in the aquatic environment have been comprehensively investigated. However, little information is available about the distribution and biogeochemical significance of these bacteria in terrestrial soil environments. In this study, stratified soils were initially examined to investigate the community structure and diversity of manganese-oxidizing bacteria. Total 344 culturable bacterial isolates from all substrata exhibited Mn(II)-oxidizing activities at the range of 1 µM to 240 µM of the equivalent MnO_2_. The high Mn(II)-oxidizing isolates (>50 mM MnO_2_) were identified as the species of phyla Actinobacteria, Firmicutes and Proteobacteria. Seven novel Mn(II)-oxidizing bacterial genera (species), namely, *Escherichia*, *Agromyces*, *Cellulomonas*, *Cupriavidus*, *Microbacterium*, *Ralstonia*, and *Variovorax*, were revealed via comparative phylogenetic analysis. Moreover, an increase in the diversity of soil bacterial community was observed after the combined enrichment of Mn(II) and carbon-rich complex. The phylogenetic classification of the enriched bacteria represented by predominant denaturing gradient gel electrophoresis bands, was apparently similar to culturable Mn(II)-oxidizing bacteria. The experiments were further undertaken to investigate the properties of the Mn oxide aggregates formed by the bacterial isolates with high Mn(II)-oxidizing activity. Results showed that these bacteria were closely encrusted with their Mn oxides and formed regular microspherical aggregates under prolonged Mn(II) and carbon-rich medium enrichment for three weeks. The biotic oxidation of Mn(II) to Mn(III/IV) by these isolates was confirmed by kinetic examinations. X-ray diffraction assays showed the characteristic peaks of several Mn oxides and rhodochrosite from these aggregates. Leucoberbelin blue tests also verified the Mn(II)-oxidizing activity of these aggregates. These results demonstrated that Mn oxides were formed at certain amounts under the enrichment conditions, along with the formation of rhodochrosite in such aggregates. Therefore, this study provides insights into the structure and diversity of soil-borne bacterial communities in Mn(II)-oxidizing habitats and supports the contribution of soil-borne Mn(II)-oxidizing bacteria to Mn oxide mineralization in soils.

## Introduction

Manganese (III, IV) oxide minerals are widely distributed in terrestrial and aquatic environments. These minerals are important in the biogeochemical cycles of a large number of metals and organic substances, and are capable of significantly affecting the transport and fate of nutrients and contaminants in the environment by catalytic and oxidative processes [1]. Increasing evidence shows that microorganisms, especially a variety of bacteria, play a dominant role in Mn(II) oxidation, which leads to the precipitation of Mn oxides in the natural systems [1], [2]. This Mn oxyhydroxide biogenesis, primarily mediated by bacteria and occurred on their surfaces via an enzymatic pathway [3], [4], is much faster than abiotic catalysis on mineral surfaces or homogeneous oxygenation in aqueous solution [5], [6].

The oxidation of soluble Mn(II) to Mn(III/IV) oxides by Mn(II)-oxidizing bacteria is presumed to be energetically favorable in storage of an electron acceptor [5]. Moreover, by encrusting themselves with Mn(III/IV) oxides, Mn(II)-oxidizing bacteria may also protect themselves from other harsh environmental conditions [1]. Previous investigations on Mn(II)-oxidizing bacteria in freshwater and marine habitats showed that most of these bacteria belonged to Alphaproteobacteria, Betaproteobacteria, and Gammaproteobacteria branches, low GC Gram-positive Firmicutes, and high GC Gram-positive Actinobacteria [1]. Three highly studied models of Mn(II)-oxidizing bacteria, namely, *Bacillus* sp. strain SG-1, *Leptothrix discophora* strain SS-1, and *Pseudomonas putida* strains MnB1 and GB-1, representing different aqueous environments have already been discussed [7]–[10]. Two soil-borne Mn(II)-oxidizing bacteria, namely, *Pseudomonas* sp. Nov. and *Citrobacter freundi* have been isolated from alfisol soil with large deposits of Mn [11]. Phylogenetically diverse bacteria have been found in Fe-Mn nodule samples and the soils surrounding these nodules [12]. In these communities, Acidobacteria- and Proteobacteria-affiliated bacteria are the dominant species. For intrinsic environments, the distribution of Mn-oxidizing bacteria in soils should be investigated to determine the diversity of the bacterial community and elucidate the formation of biological Mn oxides in the soil environment. However, very limited information is available about the mechanism of bacterial Mn oxidation taking place in soil.

Previous studies on Mn(II)-oxidizing bacteria were mainly based on different culture methods. However, culture-based methods only provide data on processes or bacterial numbers, and this kind of data is insufficient for the analysis of bacterial community composition or species diversity [13]. Small subunit ribosomal RNA (rRNA) sequence-based techniques are widely used as an alternative method to characterize bacterial communities, particularly putative manganese-oxidizing bacteria, present in ferromanganese deposits or sediments. Zhang et al. [14] investigated the bacterial community structures of soil Fe-Mn nodule samples by polymerase chain reaction (PCR) and amplified ribosomal DNA restriction analysis (ARDRA). The results showed that the identified bacteria mainly belonged to Firmicutes, Betaproteobacteria, and Gammaproteobacteria. Moreover, these bacteria were closely related to the Mn(II)-oxidizing bacteria identified from marine Fe-Mn nodules. Stein et al. [15] identified two groups related to known metal-oxidizing genera, namely, *Leptothrix* and *Hyphomicrobium* by analyzing the phylogenetic relationships of cloned 16S rRNA genes from freshwater ferromanganous micronodules and sediments. Northup et al. [16] verified the presence of some organisms in ferromanganese deposits by utilizing rRNA sequence-based methods and found that the closest relatives of such organisms are known Fe- and Mn-oxidizing/reducing bacteria. These molecular phylogenetic techniques enable the facile and recoverable detection of bacterial populations in the environment.

Terrestrial soil environments are complex and dynamic systems with soil horizons containing various bacterial communities. The characteristics of these communities differ from those thriving in aquatic environments. In this study, the composition and diversity of Mn(II)-oxidizing bacteria in stratified Fe-Mn nodule surrounding soils were examined and characterized using culture-dependent and culture-independent methods. Furthermore, variations in diversity of such bacteria in distinct soil horizons were investigated. Experiments were also conducted to investigate the combined effects of Mn(II) and carbon-rich medium on the community and micromorphological characteristics of these bacteria. The objective of this study was to ascertain the relationship between the diversity of soil-borne Mn(II)-oxidizing bacteria and their habitats as well as the biogeochemical importance of Mn oxide mineral formation in soils.

## Results

### Distribution and Phylogenetic Analysis of Highly Active Isolates

The original pH, Fe and Mn mineral contents, organic matter (OM), and cation exchange capacity (CEC) of the stratified soils were examined (Table 1). The soils were obtained from Queyu, East China and represented relatively higher pH values as well as Fe and Mn mineral contents particularly at depths of 20 cm to 75 cm. Enumeration of culturable aerobic heterotrophic bacteria of the three stratified soils shows that the B- and C-horizon soils contained similar counts (1–2×10^6^ CFU g^–1^), but these counts were less than those in the A-horizon soil (with a maximum of 5.0×10^7^ CFU g^–1^; Table 1). A total of 503 isolates from the A-, B-, and C-horizon soils were screened and their Mn(II)-oxidizing activities were determined. More than 68% of the isolates (344 in total) exhibited Mn(II)-oxidizing activity at a range of 1 µM to 240 µM of the equivalent MnO_2_ when incubated for 120 h in K medium. Considering their activities, we divided the isolates in four groups: 50 isolates were categorized with “high activity” (≥ 50 µM MnO_2_); 78 isolates with “medium activity” (10−50 µM MnO_2_); 216 isolates with “low activity” (1−10 µM MnO_2_); and 159 isolates with “no activity” (less than 1 µM MnO_2_). These isolates accounted for approximately 9.9%, 15.5%, 42.9%, and 31.6% of the total number of isolates, respectively. Notably, most of the isolates with “low activity” were present in A-horizon soil, accounting for approximately 66.2% of total “low activity” isolates in contrast to approximately 25.0% and 8.8% of B- and C-horizon soils, respectively. However, the B- and C-horizon soils harbored more “high activity” isolates by approximately 44.0% and 40% of total “high activity” isolates, respectively, in contrast to approximately 16.0% of “high activity” isolates in the A-horizon soil (Table 1).

**Table 1:** Soil characteristics at different depths.

Stratified soils	Chemical composition (means ± SD, *n* = 3)						Bacterial enumeration					
	pH value	OM^a^(g kg^−1^)	CEC^b^(cmol kg^−1^)	MnO_2_(g kg^−1^)	Fe_2_O_3_(g kg^−1^)	CaO(g kg^−1^)	Culturable cell counts (CFU^c^ g^–1^)(means ± SD, *n* = 3)	Isolates with Mn(II)-oxidizing activity offormed MnO_2_ (μM)				Total
								High (>50)	Medium (10–50)	Low (1–10)	No (<1)	
A (0 cm to 20 cm)	7.27±0.041	17.3±0.047	30.56±0.046	2.01±0.032	56.34±0.38	7.84±0.029	5.0×10^7^ (±0.047)	8	32	143	9	192
B (20 cm to 75 cm)	7.30±0.029	22.1±0.045	35.88±0.038	2.2 5±0.027	64.89±0.047	7.92±0.028	2.0×10^6^ (±0.049)	22	17	54	67	160
C (>75 cm)	7.36±0.026	7.9±0.031	45.06±0.049	0.94±0.036	63.91±0.032	12.08±0.033	1.4×10^6^ (±0.036)	20	29	19	83	151
Compiled	−	−	−	−	−	−	−	50	78	216	159	503

Note: ^a^OM, organic matter;

b CEC, cation exchange capacity;

c CFU, colony forming unit.

**Figure 1:**
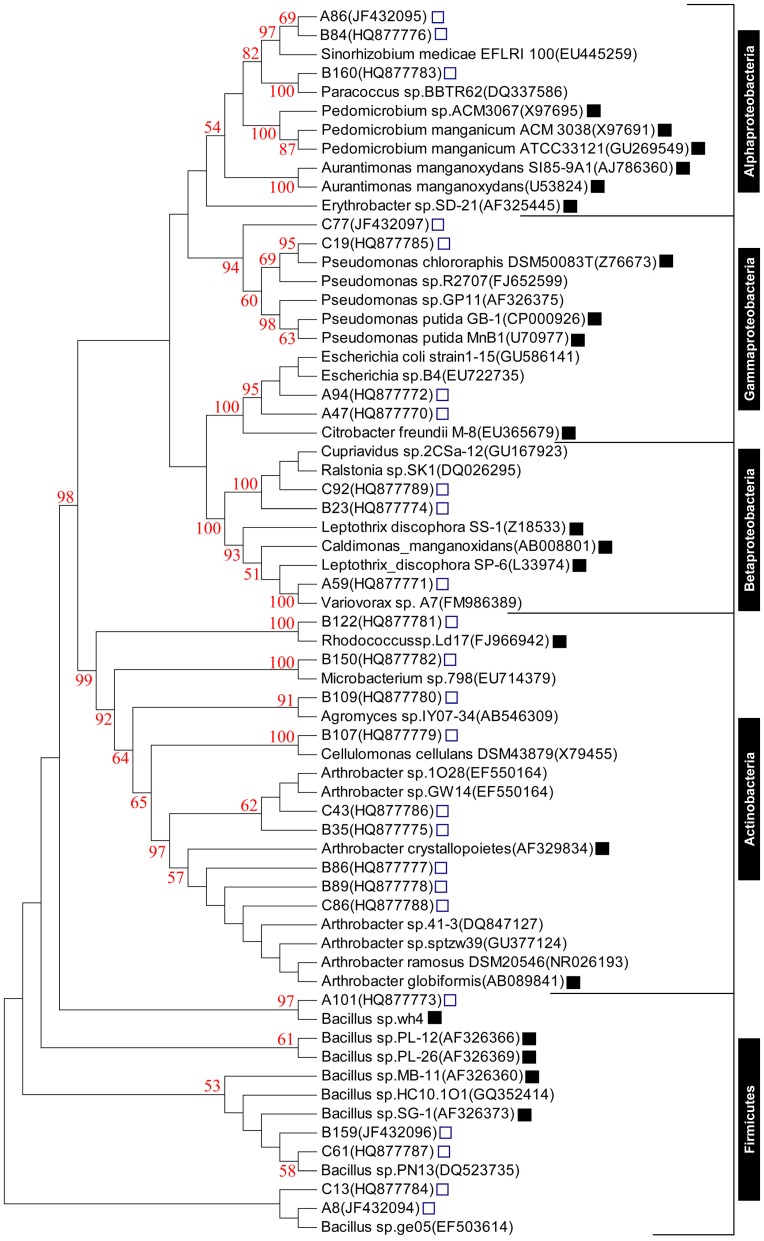
Phylogenetic relationship between the 16S rRNA gene sequences from the soil isolates with high Mn(II)-oxidizing activities (labeled with “□”) and their closest GenBank sequences with 16S rRNA gene from the known Mn(II)-oxidizing bacterial strains (labeled with “▪”) reported previously. The GenBank accession numbers of these sequences are shown in brackets. Bootstrap values ≥50% with 1,000 replicates are indicated at the branch points.

The high Mn(II)-oxidizing bacterial strains were further examined through ARDRA to find out phylogenetically related strains. A total of 24 distinct patterns were considered to represent the different strains based on *Hae*III or *Sau*3AI digestion and sequencing of the amplified 16S rRNA genes. We constructed a phylogenetic tree comprising these 24 culturable and highly active Mn(II)-oxidizing bacteria based on their 16S rRNA gene sequences (Fig. 1). The phylogenetic analysis revealed that the strains isolated from the A-horizon soil were mainly associated with Firmicutes and accounted for more than half of the total bacteria. To some extent, these strains were also associated with Proteobacteria, in which Gammaproteobacteria were dominated. Moreover, no highly active Mn(II)-oxidizing Actinobacteria strains were found in this layer. By contrast, most of the highly active Mn(II)-oxidizing bacterial strains from the B-horizon soil belonged to Actinobacteria and Alphaproteobacteria; and in the C-horizon soil, the highly active Mn(II)-oxidizing strains mainly belonged to Actinobacteria and Gammaproteobacteria (Fig. S1).

To determine the relationship among newly isolated and previously reported Mn(II)-oxidizing bacterial strains, the genera (species) with most similar sequences from GenBank, and several well-known Mn(II)-oxidizing bacteria based on the 16S rRNA gene sequences from the Ribosomal Database Project (RDP), such as *Bacillus* sp. strain SG-1, *L. discophora* strain SS-1, *P. putida* strain MnB1, *Aurantimonas manganoxydans* strain SI85-9A1, *Erythrobacter* sp. SD-21 and *Pedomicrobium* sp. ACM 3067, among others [1] were also included in the phylogenetic study (Fig. 1). The phylogenetic tree showed that the sequences could be clustered in five major branches: Actinobacteria, Firmicutes, Alphaproteobacteria, Betaproteobacteria, and Gammaproteobacteria. The distributed genera of the 24 isolates included *Arthrobacteria* (5), *Bacillus* (5), *Aurantimonas* (2), *Pseudomonas* (2), *Escherichia* (2), *Microbacterium* (1), *Cupriavidus* (1), *Variovorax* (1), C*ellulomonas* (1), *Paracoccus* (1), *Ralstonia* (1), *Rhodococcus* (1), and *Agromyces* (1). The 16S rRNA gene analysis of 24 isolates evidently revealed the presence of seven novel Mn(II)-oxidizing bacterial genera (species): *Escherichia*, *Agromyces*, *Cellulomonas*, *Cupriavidus*, *Microbacterium*, *Ralstonia*, and *Variovorax*, which have not been reported previously from any environment, for their Mn(II)-oxidation capability.

### Denaturing Gradient Gel Electrophoresis (DGGE) Analysis of Bacterial Diversity

**Figure 2:**
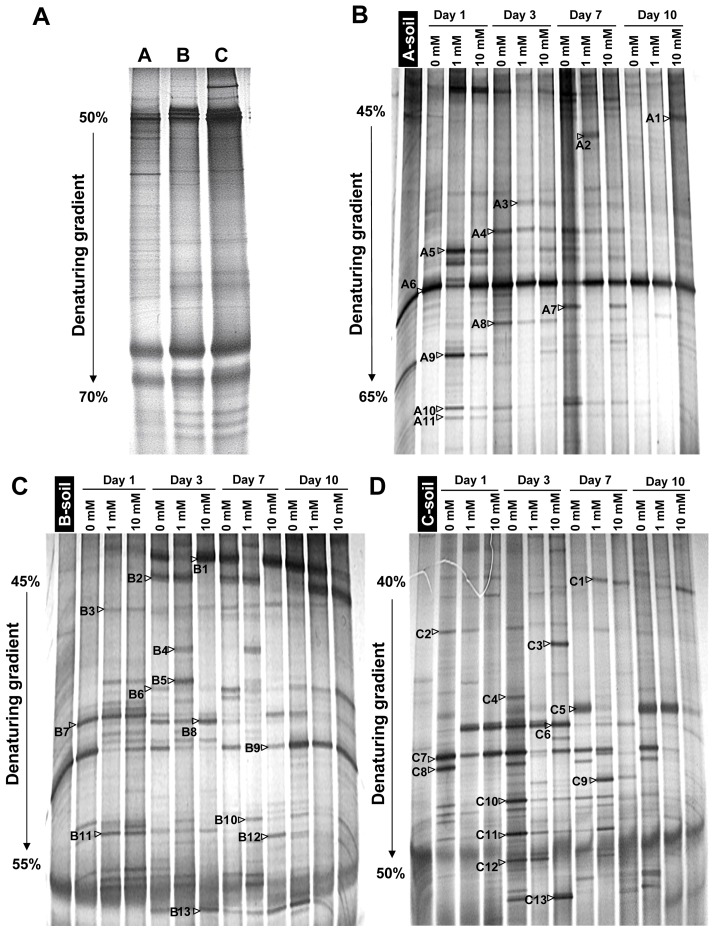
DGGE analysis of bacterial 16S rRNA V3 genes amplified from the total community DNA of the untreated soil (represented by A, B, and C labeled in lanes) and Mn (II)/carbon-rich complex medium-enriched soil (represented by 0, 1, and 10 mM labeled in lanes; from different depths; 0, 1, and 10 mM are the Mn(II) concentration). **A**: DGGE profile of the original soil from A-, B-, and C-horizon soils; **B**, **C**, and **D**: DGGE profile of Mn(II)-enriched soil from A-, B-, and C-horizon soils, respectively. Denaturant gradients of 50% to 70%, 45% to 65%, 45% to 55%, and 40% to 50% were used for the optimal separation of the products for **A**, **B**, **C**, and **D**, respectively. The numbers on the gels are the bands that were excised and sequenced corresponded to the list in Table S1.

PCR-DGGE experiments were conducted using the total bacterial 16S rRNA genes from the A-, B-, and C-horizon soils to determine the polymorphism in the untreated soils and the corresponding soils enriched with different concentrations of Mn and complex media. The DGGE profiles of the untreated A-, B-, and C-horizon soils are shown in Fig. 2A. The A-horizon soil showed more abundant electrophoresis bands compared with the other two soils, indicating that this soil layer contains a richer bacterial diversity. Fewer bands were observed in case of B- and C-horizon soils. These results were verified by calculating the Shannon index, with *H*_A = _2.72 (A-horizon), *H*_B = _1.89 (B-horizon), and *H*_C = _1.48 (C-horizon). Three DGGE gels covering a total of 39 samples of untreated and supplemented soil (with Mn(II)-and medium) extracted at different depths, were further examined to determine the effect of enrichment on bacterial diversity. All of the samples extracted from the A-, B-, and C-horizon of enriched soils, produced more abundant bands than the samples from untreated soil (Figs. 2B to 2D; Figs. S2A to S2C). However, the DGGE profiles of the soils enriched with 1 and 10 mM Mn(II) showed only limited DNA bands with the same migration distances and band densities compared with the enrichment without Mn(II). The average Shannon indexes of the diversity of the corresponding bacteria in the soils are presented in Fig. S3, in which the combined enrichments with 10 mM Mn(II) and carbon-rich medium as well as 1 mM Mn(II) and carbon-rich medium resulted in the increased diversity in the A- and B-horizon soils, respectively. However, the addition of Mn(II) did not result in corresponding increase in bacterial diversity in the C-horizon soil.

Moreover, the bacterial community structure of the A-horizon soil enriched with 0 and 1 mM Mn(II) under carbon-rich culture conditions revealed continual changes (Fig. 2B). By contrast, the bacterial community structure in soil enriched with 10 mM Mn(II) basically remained stable after 1 d of enrichment. The DGGE pattern of the B-horizon samples showed that the bacterial community structure enriched with 0 mM Mn(II) significantly changed from the 1 d to 3 d and then became primarily stable in the succeeding enrichment. Whereas the B-horizon soil enriched with 1 and 10 mM Mn(II) remained steady throughout the first 7 d of enrichment (Fig. 2C). In the C-horizon soil, no significant difference among the bacterial community structures of the enriched samples was observed except those enriched with 1 and 10 mM Mn(II) during the last 3 d of enrichment (Fig. 2D).

Thirty-seven randomly selected predominant bands from the DGGE gels of the Mn(II)-enriched A-, B-, and C-horizon soil samples were excised, purified, and sequenced for taxonomic identification. The corresponding sequences were compared with those in the GenBank database in the National Center for Biotechnology Information (NCBI) Basic Local Alignment Search *Tool* (BLAST). The matching bacterial species and phylogenetic affiliations are summarized in Table S1. The sequences mainly belonged to Gammaproteobacteria and Firmicutes. However, the response to Mn(II) enrichment differed in each soil layer. The band sequences from A-horizon gels mainly corresponded to Firmicutes and Gammaproteobacteria. The band sequences from the B-horizon gels were primarily related to Gammaproteobacteria and unclassified bacteria. The C-horizon gel sequences also possibly belonged to Gammaproteobacteria. However, not even a single species of Actinobacteria was found.

### Multidimensional Scaling (MDS) Analysis of DGGE Patterns and Sequence Analysis of DGGE Bands

MDS analysis was performed using the DGGE banding pattern to investigate the changes in the bacterial community structure during enrichment and illustrated the similarity of the possible pairs of each gel track. A two-dimensional plot of MDS scores for A-, B-, and C-horizon soils is illustrated in Fig. S4. The positive and negative values displayed along the *X* and *Y* axes of the figures are simply used for plotting purposes and the scales are unsuitable for the comparison of different figures. The configurations indicated the presence of a central core data group represented by the points inside the dotted circle of the A-, B-, and C-horizon soils. The samples enriched for 3 d (D3) and 7 d (D7) were found within the same central core in each soil horizon, suggesting that the enrichments from D3 to D7 did not result in a remarkable variation in bacterial diversity. By contrast, the untreated soil samples represented by “◊” were far from the central core group, indicating that the enrichment significantly affected bacterial diversity.

### Kinetic Examination, Scanning Electron Microscope (SEM)/Energy-dispersive X-ray Spectroscopy (EDX) Assays of Mn(II)-oxidizing Bacteria and their Manganese Oxides

**Figure 3:**
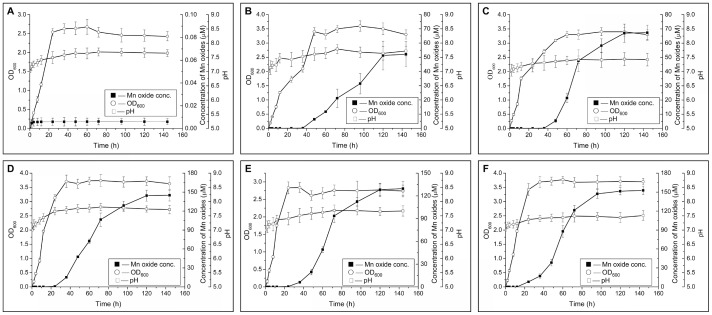
Time course of the Mn(II)-oxidizing activities and the cell growth of several isolates from A-, B-, and C-layer soils. The cells were grown in liquor K medium for 144(OD_600_) and the concentration of Mn oxides were determined according to the procedures described in the “Materials and Methods” section. (○) Optical density of the cells (at 600 nm); (▪) Concentration of Mn oxides; (□) pH. A: Non-Mn(II)-oxidizing *E. coli* JM109 (as the negative control). **B**: A86. **C**: A101. **D**: B84. **E**: C19. **F**: C13.

The 24 isolates with high Mn(II)-oxidizing activities were cultured in a laboratory shake-flask trial to determine the biological Mn(II) oxidation and micromorphological response of Mn(II)-oxidizing bacteria after a continuous Mn(II) and carbon-rich enrichment process. The Mn(II)-oxidizing activities of the five isolates, including A86 and A101 from the A-horizon soil, B84 from the B-horizon soil, and C19 and C13 from the C-horizon soil were kinetically examined to verify the biotic oxidation of Mn(II) to Mn(III/IV), forming Mn oxides under such culture conditions. The concentration of Mn oxide continuously increased with the culture time-course (144 h) of these isolates. This result differed from the low Mn oxide level of the control strain *E. coli* JM109 (Fig. 3). In addition, the pH levels of the culture suspensions were maintained at 7.2 to 7.8 (Fig. 3) and reached a stable level at <8.0 during the three-week culture process (data not shown). Therefore, these results demonstrated the biological Mn(II) oxidation of these isolates and, noteworthy, the oxidation was started at stationary phase by these isolates.

**Figure 4:**
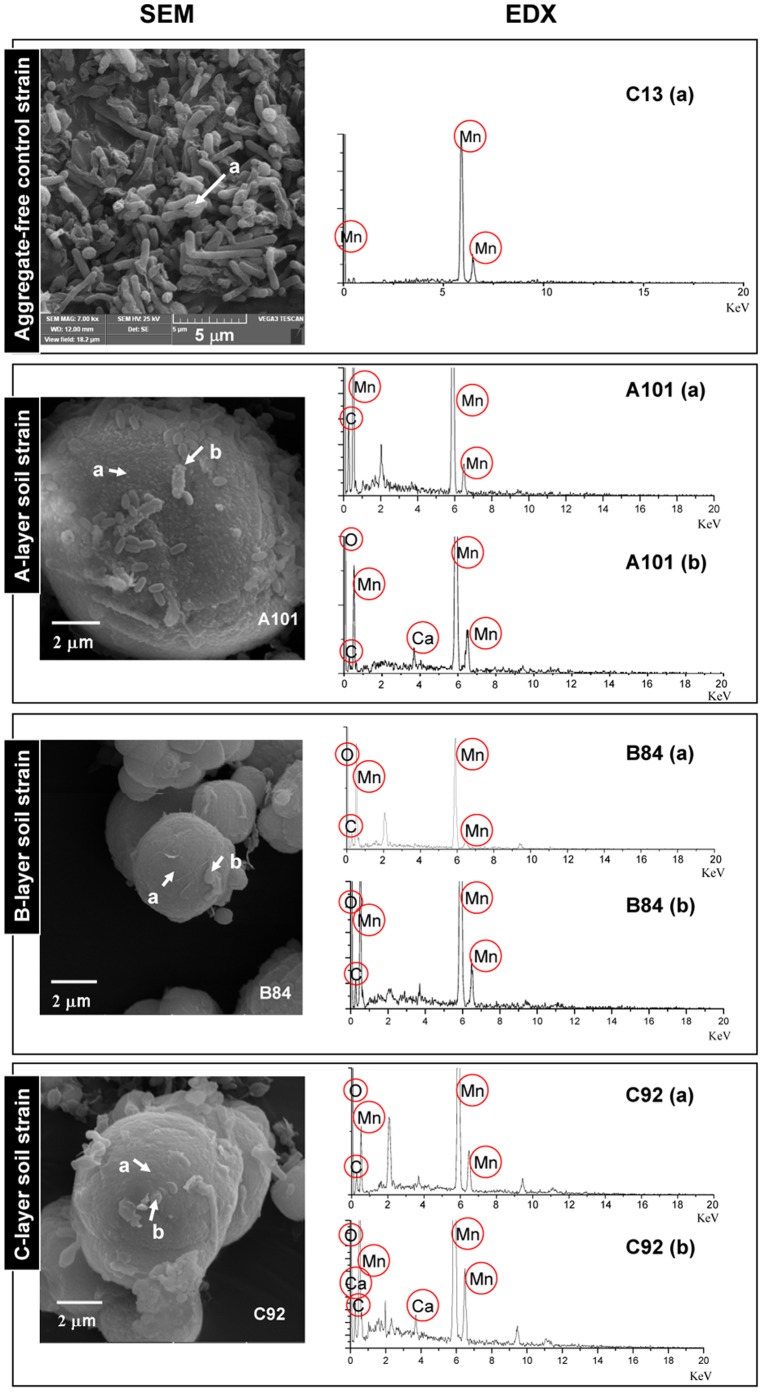
SEM images of the mixture of bacteria and Mn oxides as well as EDX spectra of the selected areas. In SEM, A101, B84, and C92 were the isolates from the A-, B-, and C-layer substrata, respectively, illustrating the formation of Mn oxide aggregates; C13 represents an SEM image of an isolate having no capability to produce Mn oxide aggregates. Two scanning areas for EDX analysis in the SEM images of A101, B84, and C92 were indicated by a, b, and arrows, respectively. A single scan was indicated for C13.

SEM revealed that 18 isolates formed regular microspherical aggregates in the three-week culture process. The diameters of the aggregates varied from approximately 10 µm to 20 µm. The images of micromorphologies of the isolates from A- (A101, A86), B- (B84, B159), and C-horizon (C92, C19) soils taken by SEM, are shown in Fig. 4 (A101, B84, and C92) and Fig. S5 (A86, B159, and C19). In these images, the attached and embedded bacteria around the aggregates can be easily distinguished. However, some isolates did not form such aggregates; for example, the isolate C13 exhibited Mn(II)-oxidizing activity (Fig. 3F) but failed to form aggregates (Fig. 4A). The EDX scanning assay of the microspherical aggregate surfaces showed that Mn is one of the main elements in addition to C and O that cover the surfaces of the aggregates and the bacteria (Figs. 4 and S5).

**Figure 5:**
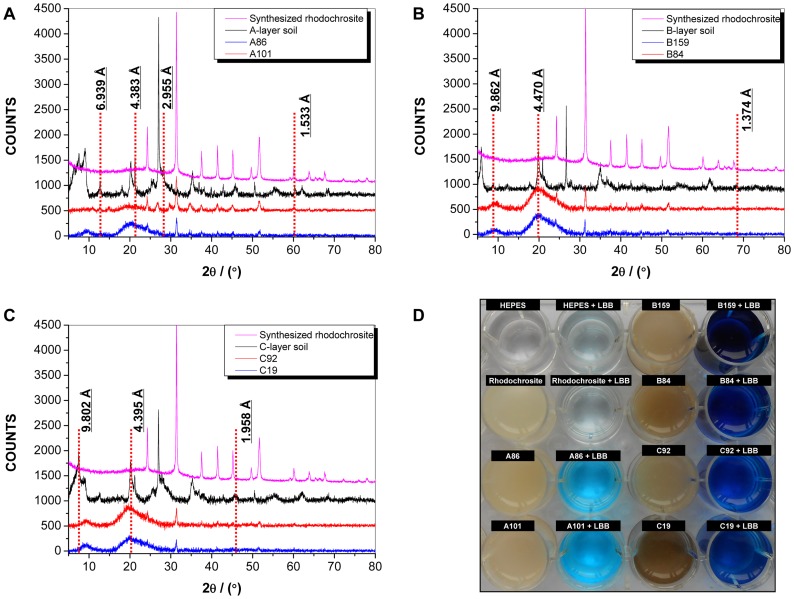
XRD patterns (A, B, and C) and LBB tests (D) of Mn oxides from different depths of soils and Mn oxides from different Mn(II)-oxidizing bacteria. The experiments were performed using dried powdered Mn oxide samples. In A, B, and C, the red dashed lines indicate the overlapping peaks; In C, a HEPES buffer and a synthesized rhodochrosite sample were used as the negative controls.

X-ray diffraction (XRD) assays were conducted to verify the types of Mn oxides from the microspherical aggregates and soil samples. The XRD profile of the microspherical aggregates formed by the Mn(II)-oxidizing bacteria from all of the three soil layers, showed distinct peaks of rhodochrosite (JCPDS 83–1763; Figs. 5A to 5C). However, along with the rhodochrosite, the peaks of sodium manganese (JCPDS 32–1126), potassium manganese oxide (JCPDS 36–0873), potassium sodium manganese oxide (JCPDS 81–0284), ramsdellite (JCPDS 44–0142), sidorenkite (JCPDS 33–1266), and δ-MnO_2_ (JCPDS 44–0141) were also found. The presence of such Mn oxides was also observed in the results of the leucoberbelin blue (LBB) tests of the samples prepared from the microspherical aggregates based on the substantial Mn(II)-oxidizing activity in contrast to the negative activity of a synthesized rhodochrosite sample (Fig. 5D). Some of the overlapping peaks from each microspherical aggregate and soil sample at the same stratum were verified. In the A-layer, the peaks at 0.6939, 0.4383, 0.2955, and 0.1533 nm overlapped (Fig. 5A). The peaks at 0.9862, 0.4470, and 0.1374 nm overlapped in the B-layer (Fig. 5B). The peaks at 0.9802, 0.4395, and 0.1985 nm overlapped in the C-layer soil (Fig. 5C).

## Discussion

Most of the Mn(II)-oxidizing bacteria have been isolated from marine or other aquatic environments, only few investigations have been conducted in terrestrial environments and, moreover, a Mn(II)-oxidizing gene (cluster) similar to that of marine bacteria [7] has not been identified yet in soil-borne bacteria. Thus, the community composition and diversity of soil-borne Mn(II)-oxidizing bacteria needs to be investigated. For this purpose, various culturable strains should be obtained for the mechanistic studies of bacterial Mn(II) oxidation in soils. In this study, soil samples were obtained from a plot where a spot of scattered Fe-Mn nodules was found [the average MnO_2_ and Fe_2_O_3_ contents were 1.73 and 61.71 g per kg soil, respectively (Table 1)]. The soil samples were used to study the diversity of Mn(II)-oxidizing bacterial community and to isolate bacterial strains. Soil samples were found to be rich in Mn(II)-oxidizing bacterial strains. The plate culture showed that the bacteria exhibiting Mn(II)-oxidizing activities accounted for more than half of all the isolates, indicating the abundance of culturable Mn(II)-oxidizing bacteria in this soil plot. Moreover, it is apparent that the distribution of Mn(II)-oxidizing bacteria was correlated with the depth of the soil (Table 1). Although most of the Mn(II)-oxidizing isolates were obtained from the A-stratum soil, the isolates with high Mn(II)-oxidizing activities (>50 mM MnO_2_) were dominant in B and C strata. In view of different environmental conditions, especially in terms of Mn^2+^/Fe^3+^, OM, and CEC levels in these strata, we presumed that the distribution variance of Mn(II)-oxidizing bacteria in these strata might be a consequence of evolutionary adaptation of microbial community to environmental conditions, as in the upper A-stratum soil, the relatively low total Mn^4+^/Fe^3+^-oxide level and the accumulation of organic matters resulted from bioclimatic and human activities, might allow the abundant growth of various bacteria, whereas in the B- and C-horizon soils, the relatively higher total Mn^4+^/Fe^3+^-oxide level was possibly more surmountable for Mn(II)-oxidizing bacteria, especially those with high Mn(II)-oxidizing activity. It is of interest to further investigate whether Mn^2+^/Fe^2+^ is favorable or endurable for Mn(II)-oxidizing bacteria, possibly for chemolithoautotrophic growth by oxidizing Mn^2+^/Fe^2+^ or developing protective mechanisms [5].

Sequence analyses of 16S rRNA genes of culturable bacteria with high Mn(II)-oxidizing activities enable the fast and fundamental identification of these soil-borne bacterial species. The results revealed that these bacteria belonged to Actinobacteria, Firmicutes, Alphaproteobacteria, Betaproteobacteria, and Gammaproteobacteria (Fig. S1). This result is consistent with the previous taxonomic classification of marine Mn(II)-oxidizing bacteria [1]. The dominant bacteria detected in the A-horizon soil belong to Firmicutes mainly comprising *Bacillus*, which is known to undergo Mn(II) oxidation [17]. However, the B-horizon and C-horizon soils showed significantly different Mn(II)-oxidizing bacterial patterns from that of the A-horizon soil; most of the high Mn(II)-oxidizing isolates from the deeper B- and C-horizon soils belong to Actinobacteria. Moreover, a high ratio of previously uncharacterized Mn(II)-oxidizing genera (species) (7 of 24 isolates) was observed (Fig. 1). The presence of Mn(II)-oxidizing Actinobacteria and new Mn(II)-oxidizing genera (species) indicated the variation in soil and marine habitats of Mn(II)-oxidizing bacteria [17], [18]. Keeping in view the abundant distribution of Actinobacteria bacteria in soil, we propose that soil-borne Mn(II)-oxidizing Actinobacteria and the newly characterized Mn(II)-oxidizing species, might be thermodynamically favorable for the oxidation of Mn(II) and are essential for the biogeochemical cycle of Mn in the soil environments.

In a laboratory trial, the effect of Mn(II) on stratified soils at a concentration range of 0 mM to 10 mM, was determined to evaluate the response of the bacterial community structure and diversity to environmental Mn(II) stress. To facilitate the growth of the isolates, we used a complex medium with a higher dose of carbon source than the soils. Therefore, the Mn(II) enrichments under the specific culture conditions rather than in natural soil environments were evaluated. The results presented in this study demonstrated that Mn(II) caused a substantial increase in bacterial diversity in A- and B-horizon soils but not in C-horizon soil (Figs. 2B to 2D). This observation may be explained by the habitat-specific tolerance of bacterial species to alone Mn(II) content, where the A-horizon soil bacteria were very tolerant to higher Mn(II) concentrations, and the tolerance of the B- and C-horizon soil bacteria gradually decreased. Noticeably, both A- and B-horizon soils had higher alone MnO_2_ background contents compared to relatively low alone MnO_2_ value of the C-horizon soil as shown in Table 1. Thus, difference in tolerance of the isolates from different soil stratum may be the consequence of environmental adaption in terms of different MnO_2_ background contents.

Abiotic oxidation of Mn(II) occurs preferentially at pH >8.0 [19], [20]. Moreover, anaerobic conditions are favorable for the formation of some Mn compounds such as rhodochrosite (MnCO_3_) [21] or MnS [22], [23]. In the current study, the enrichment of Mn(II) and carbon-rich complex medium resulted in increased bacterial population and similar phylogenetic classifications to those of culturable Mn(II)-oxidizing bacteria (Fig. S2). Thus, the Mn(II) oxidation conditions of these isolates should be investigated under such enrichment conditions. The Mn(II) oxidation of several isolates were kinetically examined and showed substantial Mn(II)-oxidizing activities during the time course at pH <8.0 (Fig. 3). Therefore, Mn oxidation apparently occurred through biotic transformation process. However, the formation of rhodochrosite (Fig. 5) in all tested strains under the enrichment conditions reflects the complex metabolic pathways. It has been well known that Mn(II) could be converted to other forms, such as MnO_2_, Mn(OH)_2_ or MnCO_3_, under different Eh and pH conditions. MnCO_3_ have been reported to idiomorphically occur in pH>8, CO_3_^2−^>4.4×10^−3^ mM and Eh<418 mV [24] and are stable under reducing (Eh = −200 to −600 mV) and alkaline (pH = 8–10) conditions [23]. Moreover, some Mn(II)-oxidizing bacteria have been shown to reduce the Mn oxides under low oxygen or anaerobic circumstances either enzymatically or non-enzymatically [25]. In this study, the relatively higher CO_3_^2−^ concentration resulted from carbon-rich culture condition, and potentially anaerobic microenvironment might be produced both intracellularly and extracellularly as a result of increased cell density particularly at the later culture stage, are all preferentially contributed to the formation of rhodochrosites. Although the exact mechanism is yet unclear, we proposed the both, abiotic transformation of Mn(II) to MnCO_3_ and biogenetic reduction of Mn(III/IV) to Mn(II) and MnCO_3,_ could possibly have occurred simultaneously, which resulted in the accumulation of rhodochrosites.

Although isolates from different substrata exhibited morphological diversity, many isolates with high Mn(II)-oxidizing activities formed regular microspherical aggregates upon continuous Mn(II)- and complex medium-enrichment in a laboratory shake-flask trial. Kinetic examinations, XRD assays, and LBB tests reveal that such formation indicates the Mn oxide component of the aggregates or at least a certain amount of Mn oxides in such aggregates should be considered. Moreover, SEM examination showed that only some of the Mn(II)-oxidizing bacterial isolates were able to form microspherical aggregates. This suggests that these aggregates were most likely formed by host strain-specifically biotic Mn(II) oxidation but not non-specific abiotic process. The difference in aggregate sizes may be attributed to the different Mn(II)-oxidizing activities of various isolates. To the best of our knowledge, this study is the first to report a distinctive micromorphological characteristic resulting from Mn(II)- and carbon-rich enrichment of the soil-borne Mn(II)-oxidizing bacteria. This result can be associated with certain correlations between microspherical aggregates and Mn oxide minerals as well as ferromanganese nodules in soil. The biofilm formed by the microflora was observed in manganese/polymetallic nodules from the Clarion-Clipperton zone in the Eastern Pacific Ocean basin [26]. The oxidation reactions involving the Mn(II)-oxidizing bacteria occurred on bacterial surfaces [27]. Therefore, microspherical aggregates might be formed due to the precipitation of Mn oxides surrounding the Mn(II)-oxidizing bacteria. Thus, the formation of aggregates can be closely associated with the formation of Mn oxide minerals and/or the ferromanganese nodules in superficial environments under the combined effects of various microorganisms and chemical reactions with different elements.

The XRD patterns of the Mn oxides produced under laboratory conditions were partially similar to those of the soils (Fig. 5). Moreover, the amount of Mn oxides produced in the laboratory conditions, was also less than natural conditions. Difference in the conditions of both environments (laboratory and natural) could be the possible reason for these variations. For example, the laboratory experiment conducted to investigate the Mn(II) oxidation, was carried out on pure bacterial isolates whereas in natural environment, a diverse microflora might be responsible for biotic oxidation of Mn. Moreover, the chemistry of natural environment differs a lot from laboratory conditions. Effects of these variations on Mn oxidation could not be rule out. Considering that a complete simulation of the complex conditions of natural soil environment is impossible, it was believed that Mn oxides produced under laboratory conditions, could only be few representatives of various environmental Mn oxide minerals. However, it needs to be further investigated that whether the Mn oxides tested in the laboratory, can be converted to soil Mn oxide minerals or not. Furthermore, the contributions of different substrata on overall diversity and compositions of soil Mn oxide minerals should be investigated as different types of oxides were produced from various substrata.

## Conclusion

This study examined the bacterial composition and diversity patterns of Mn(II)-oxidizing bacteria in a terrestrial soil environment by using culture-dependent and culture-independent methods. Culturable Mn(II)-oxidizing bacteria were found to abundantly distribute in sampling soils. Mn(II)-oxidizing bacteria with relatively high activities were primarily distributed in the deeper B- and C-horizon soils and mainly belonged to Actinobacteria, Firmicutes, Alphaproteobacteria, Betaproteobacteria, and Gammaproteobacteria. The increase in the diversity of soil bacterial community was observed after the combined enrichment with Mn(II) and carbon-rich complex medium. The phylogenetic classification of the enriched bacteria was apparently similar to those of culturable Mn(II)-oxidizing bacteria. In laboratory experiments, some bacteria isolates with high Mn(II)-oxidizing activity were closely encrusted with their Mn oxides and formed regular microspherical aggregates under prolonged Mn(II) and carbon-rich medium enrichment for three weeks. The result indicated that formation of microspherical aggregates could be associated with Mn oxide mineralization in natural soil environment.

## Experimental Procedures

### Soil Sampling

In November 2008, soil samples were collected from a state owned uncultivated land in Queyu, Shandong Province, East China. The field study, including sampling and analysis had been approved by the National Natural Science Foundation Council of China with the issue of a grant (item no. 40830527). The soil contained scattered Fe-Mn nodules and the sampling procedure described by Soil Science Society of America (https://www.soils.org/lessons/teachers-guide/soil-formation) was followed. Briefly, three stratified soil samples were collected from the surface soil layer (at a depth of 0 cm to 20 cm), the subsoil layer (at a depth of 20 cm to 75 cm), and the substratum (at a depth >75 cm). These layers were designated as the A-, B-, and C-horizon soils, respectively. Each stratum sample was collected from five randomly selected spots, vigorously mixed, and filtered using an autoclaved sieve with 1 mm sieve pores to remove the Fe-Mn nodules. All of the soil samples were stored in separate sterile sample bags placed in an ice bath, transported to the laboratory, and stored at 4°C for approximately one week until they were used in the experiments.

### Soil Chemical Analyses

Soil chemical analysis was conducted using the air-dried soil samples. The soil pH was measured from a soil suspension (1∶2.5 in deionized water) by using a pH meter. The soil OM was determined using the K_2_Cr_2_O_7_ oxidation method [28]. The CEC was determined using the ammonium chloride method [29]. Soil elemental analysis was conducted as previously described [30]. For the detection of hematite concentration, procedure described by Tan et al. [30] was followed. Briefly, soil samples were screened with 60 mesh and samples were suspended in 0.1 M HAHC (hydroxylamine hydrochloride) in a ratio of 1∶ 100. Suspension was mixed for 2 hours at 25°C and then, subjected to centrifugation. Supernatant was used for the analysis of Mn and Fe contents using atomic absorption spectroscopy.

### Isolation, Culture, and Enumeration of Soil Bacteria

The isolation and enumeration of culturable bacteria from the soil samples were conducted based on established standard protocols [31]. The bacteria were cultured at 30°C, in beef peptone (BP) medium plates containing (per liter of deionized water) 5 g of beef extract (Difco), 10 g of Difco Bacto-peptone, 5 g of sodium chloride, and 15 g of Difco Bacto agar (pH 7.2 to 7.4), unless otherwise specified. The enumeration of plate CFUs was performed in triplicate, and the results were presented as the mean of triplicate assays.

### Mn(II)-oxidizing Activity Assay and LBB Test

All of the isolates were grown in a liquid BP medium (5 mL) for 24 h. A total of 50 µL of each culture was harvested by centrifugation. The pellet was transferred to 5 mL of liquid K medium [32] containing FeSO_4_·7H_2_O (0.001 g/L), MnCl_2_ (0.2 g/L), peptone (2.0 g/L), yeast extract (0.5 g/L), and 10 mM HEPES buffer (*N*-2-hydroxyethylpiperazine-*N*′-2-ethanesulfonic acid; pH 7.5). To quantitatively determine the Mn(II)-oxidizing activities of the isolates, we measured the oxidized Mn content produced (primarily assumed to be MnO_2_) in the cell suspension after 120 h of growth in liquid K medium by using leucoberbelin blue (Sigma-Aldrich) method as described by Krumbein and Altimann [33]. KMnO_4_ was used as a standard to read the absorbance at 620 nm. The amount of MnO_2_ produced (1 µM MnO_2_ corresponds to 0.4 µM KMnO_4_) was defined as the Mn(II)-oxidizing activity of the bacterial cells and it was further categorized as “high activity” (≥50 µM MnO_2_ per liter of culture), “medium activity” (10–50 µM MnO_2_), “low activity” (1–10 µM MnO_2_), and “no activity” (<1 µM MnO_2_). The kinetic examination of Mn(II)-oxidizing activity of the isolates was performed by analyzing the concentration of Mn oxides, present in the cell suspension. For this purpose, samples were taken in a specific time course and analyzed by above stated procedures. The freeze-dried aggregate powders prepared by XRD assay procedures [34] were qualitatively analyzed by LBB test according to a previously described method [33] where 1 mM MnCl_2_ (final concentration) was added to the reaction solution.

### DNA Extraction, PCR Amplification, and ARDRA

The total DNA from the soils was extracted using a Biospin gel extraction kit (Bio-Rad, CA, USA). To extract DNA from Mn(II)-enriched soils, 1 g of soil was added in K_1_ medium (K medium with a final concentration of 1 mM MnCl_2_), K_10_ medium (K medium with a final concentration of 10 mM of MnCl_2_), and K_0_ medium (K medium without the addition of MnCl_2_ however, it contained small amount of Mn after the soil was added for enrichment because of the presence of natural Mn content in soil). The resulting soil mixture in the medium was incubated at 30°C for 1, 3, 7, and 10 d. The soil suspensions were then subjected to DNA extraction by using the previously mentioned kit. The total DNA from the cultured bacterial cells was extracted based on standard procedures [35].

Bacterial 16S rRNA genes of the culturable isolates were amplified by PCR using bacterial-specific primers (27F/1492R) according to the method described by He et al. [12] with slight modifications. For the PCR amplification of the V3 segment of the 16S rRNA genes that were extracted from the soils, the nested PCR was conducted using primers 27F and 1492R [12] as well as 338F (5′-CCTACGGAGCAGCAG-3′) and 518R (5′-ATTACCGCGGCTGCTGG-3′) spanning 180 bp of the V3 region of the bacterial 16S rRNA genes [36]. A 40 bp GC clamp (CGC CCG CCG CGC GCG GCG GGC GGG GCG GGG GCA CGG GGG G) was added to the primer 338F to increase the DNA band separation in the DGGE gel [37].

Among the numerous isolated bacteria, those with high Mn(II)-oxidizing activities were selected for ARDRA. The PCR-amplified 16S rRNA genes were digested with *Hae*III and the ARDRA patterns were grouped. The isolates with similar patterns were further digested and regrouped with *Sau*3AI. One isolate from each group was sequenced.

### DGGE Analysis

The total DNA of A-, B-, and C-horizon soils were used as templates for the PCR amplification of the 16S rRNA gene V3 segments, which were detected using DGGE in 25 µL aliquots per lane, by using a D-Code universal mutation detection system (Bio-Rad, CA, USA). Electrophoresis was conducted in 8% (w/v) acrylamide gel with 45% to 70% denaturing gradient in 1× TAE buffer (20 mM Tris acetate, 10 mM sodium acetate, and 0.5 mM EDTA) at a constant voltage of 200 V at 60°C for approximately 5 h. The gel was stained by silver staining, destained with deionized water, scanned, and analyzed using the Gel Doc 2000 Quantity-One 4.5.2 gel documentation system (Bio-Rad, CA, USA) according to the manufacturer’s protocol to generate a densitometric profile. The resultant bands detected at a ratio of the relative peak height to the total peak height greater than 1% were analyzed in two ways as follows:

(i) The Shannon index of bacterial diversity *H* [38] was used to determine the diversity of the taxa present in the original and enriched soil samples. This index was calculated using the following equation:
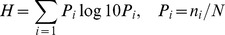
where *P_i_* represents the significant probability of the bands in a gel lane, *n_i_* represents the height of a peak, and *N* is the sum of the band peak heights in the densitometric profile.

(ii) The similarity of the banding pattern among each gel lane was calculated to establish the distance matrix in each gel lane by using the following function:
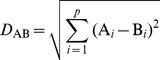
where *A_i_* represents the significant probability of each band in gel lane A and *B_i_* represents the significant probability of each band in gel lane B. No band was treated as zero; *P* = (total number of bands on lane A)+(total number of bands on lane B) – (total number of bands common to lane A and lane B). The distance matrix was analyzed using MDS analysis [39] in SPSS 17.0 software. The resulting graphical representation, MDS map (showing every band pattern as one plot), and relative changes in the community structure can be visualized as the distance between plots. The DGGE banding patterns are more similar when the plots are closer to each other.

The predominant DGGE bands were excised from the gels and used as templates to amplify the 16S rRNA gene-V3 segments by using the 338F and 518R primer pairs without the GC clamp. The products were then purified using a PCR purification kit (QIAGEN) and cloned in a pMD18-T plasmid vector system (TaKaRa, Japan) for sequencing.

### Sequencing and Phylogenetic Analysis

The DNA bands of the corresponding isolates with different ARDRA patterns and the predominant DGGE bands were excised from the gels, purified, and sequenced. Sequence alignments were performed using the Clustal X software [40], and those that differed by <3% were grouped in the same phylotype. Each sequence representing one phylotype was analyzed using the Chimera Check program of the RDP database (http://rdp.cme.msu.edu) [41] to exclude the chimeric artifacts. Each sequence was also searched in the NCBI GenBank database by using the BLAST program.

The GenBank sequences that were most similar to the isolates were collected for phylogenetic tree construction based on the Kimura two-parameter model and the neighbor-joining algorithm in MEGA version 5.2 (http://www.megasoftware.net/mega.php). Bootstrap analysis with 1,000 replicates was performed to assign the confidence levels to the tree nodes. The sequences were submitted to the GenBank database to obtain a series of accession numbers from HQ877770 to HQ877789 and from JF432094 to JF432097. The GenBank sequences that were most similar to the predominant DGGE bands were collected for tentative identification. The determined sequences of these DGGE bands were also submitted to the GenBank database to obtain accession numbers from HQ877790 to HQ877815 and from JF432098 to JF432108.

### SEM/EDX Assays

The isolates with high Mn(II)-oxidizing activities were grown in liquid K medium containing 1 mM Mn(II). The overnight-grown (∼ 13 h) inoculum was transferred (1% vol/vol) to 100 mL of K medium [containing 1 mM Mn(II)] in a 500 mL Erlenmeyer flask at 30°C in an air-shaking incubator at a gyration rate of 150 rpm and incubated for three weeks. The suspensions were then centrifuged and prepared for SEM analysis according to a previously described method [42]. SEM observation and EDX assay were conducted using JSM-6390/LV SEM (NTC, Japan) equipped with an EDX spectral detector.

### XRD Analysis

Soil and bacterial Mn oxides were extracted according to previously described procedures [34]. The dried oxides were ground to obtain powdered samples. XRD measurements were conducted using a Bruker D8 Advance diffractometer equipped with a Lynx-Eye detector by Ni-filtered Cu K_α_ radiation (λ = 0.15406 nm). The diffractometer was run at a tube voltage of 40 kV and a tube current of 40 mA with a scanning rate of 1°/min at a step size of 0.02°.

### Statistical Analysis

All the assays were performed in triplicate and data represents the mean values. Statistical analysis was conducted using SPSS version 17.0 software. Statistical significance was defined at *P*<0.05.

## Supporting Information

Figure S1 Phylum-level phylogenetic diversity and abundance of the 16S rRNA gene sequences from culturable bacteria with high Mn(II)-oxidizing activities. Sequences were assigned to their respective phyla by using the RDP classifier software.
(TIF)

Figure S2 Illustration of the total amplified bands on the DGGE profile shown in Fig. 2. A, B, and C correspond to Figs. 3B, 3C, and 3D, respectively.
(TIF)

Figure S3 Average diversity index of Mn(II)-enriched soil samples calculated from **B**, **C**, and **D** in Fig. 5.
(TIF)

Figure S4 Two-dimensional plots of the MDS analysis results of the DGGE patterns of Mn(II)-enriched soil samples from different depths (represented by A, B, and C) in Figs. 2**B, C, and D**. ◊, original soil; •, 0 mM Mn(II)-enriched soil; ▪, 1 mM Mn(II)-enriched soil; and ▴, 10 mM Mn(II)-enriched soil.
(TIF)

Figure S5 SEM images of the mixture of bacteria and Mn oxides as well as EDX spectra of the corresponding selected areas. A86, B159, and C19 illustrate the formation of Mn oxide aggregates; C13 represents a sample without the formed Mn oxide aggregates.
(TIF)

Table S1 Summary of the 16S rRNA gene sequences obtained from the predominant bands in the DGGE gel from different soil horizons and the closest match to the NCBI nucleotide sequence database (GenBank)^a^.
(DOC)
