# Supplementary figures and images for: Population Structure of Manganese-Oxidizing Bacteria in Stratified Soils and Properties of Manganese Oxide Aggregates under Manganese–Complex Medium Enrichment
Source: PLoS One. 2013 Sep 12;8(9):e73778. doi: 10.1371/journal.pone.0073778 (PMC3772008; doi:10.1371/journal.pone.0073778)

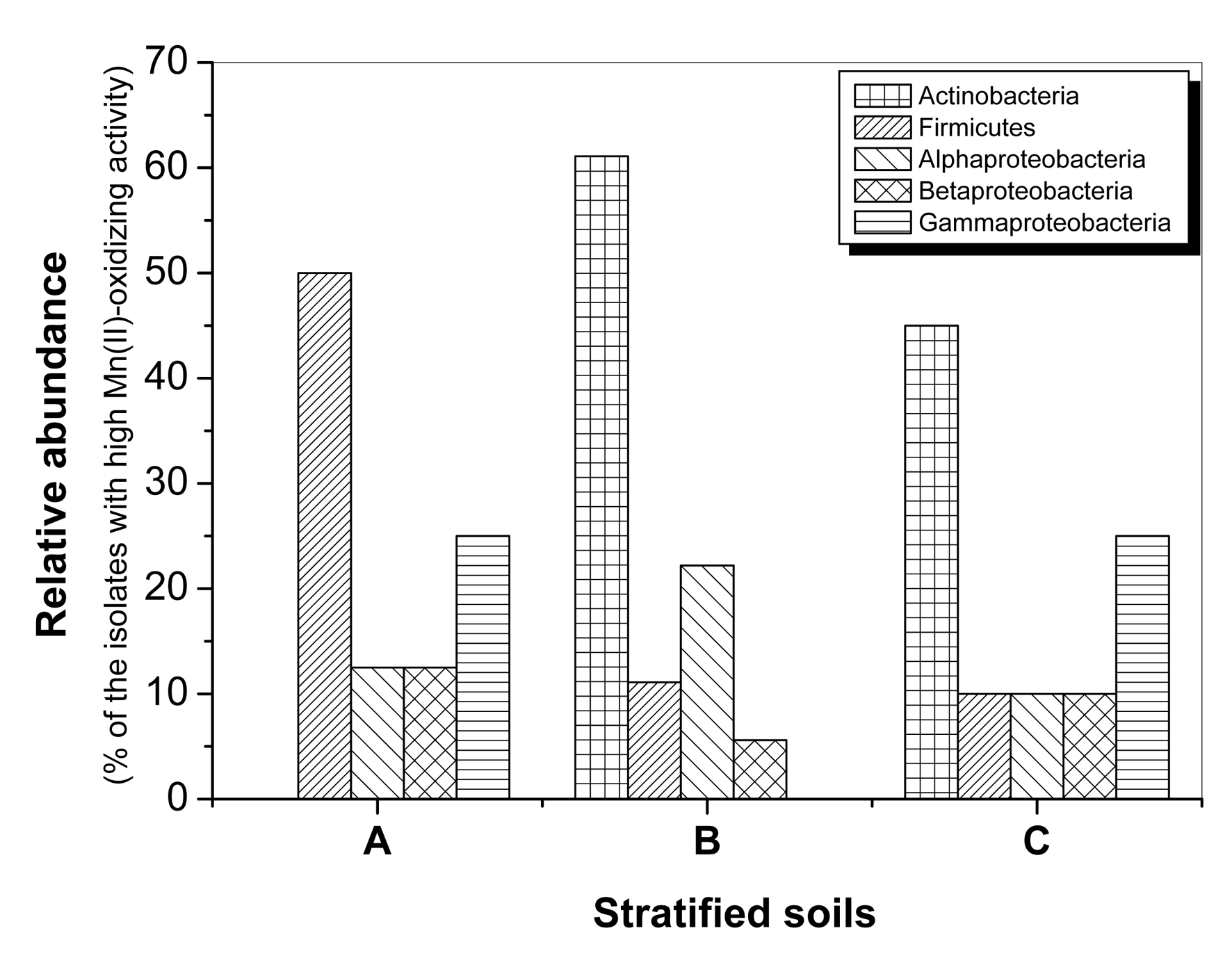

Supplement: Figure S1 — Phylum-level phylogenetic diversity and abundance of the 16S rRNA gene sequences from culturable bacteria with high Mn(II)-oxidizing activities. Sequences were assigned to their respective phyla by using the RDP classifier software. (TIF) [file pone.0073778.s001.tif]

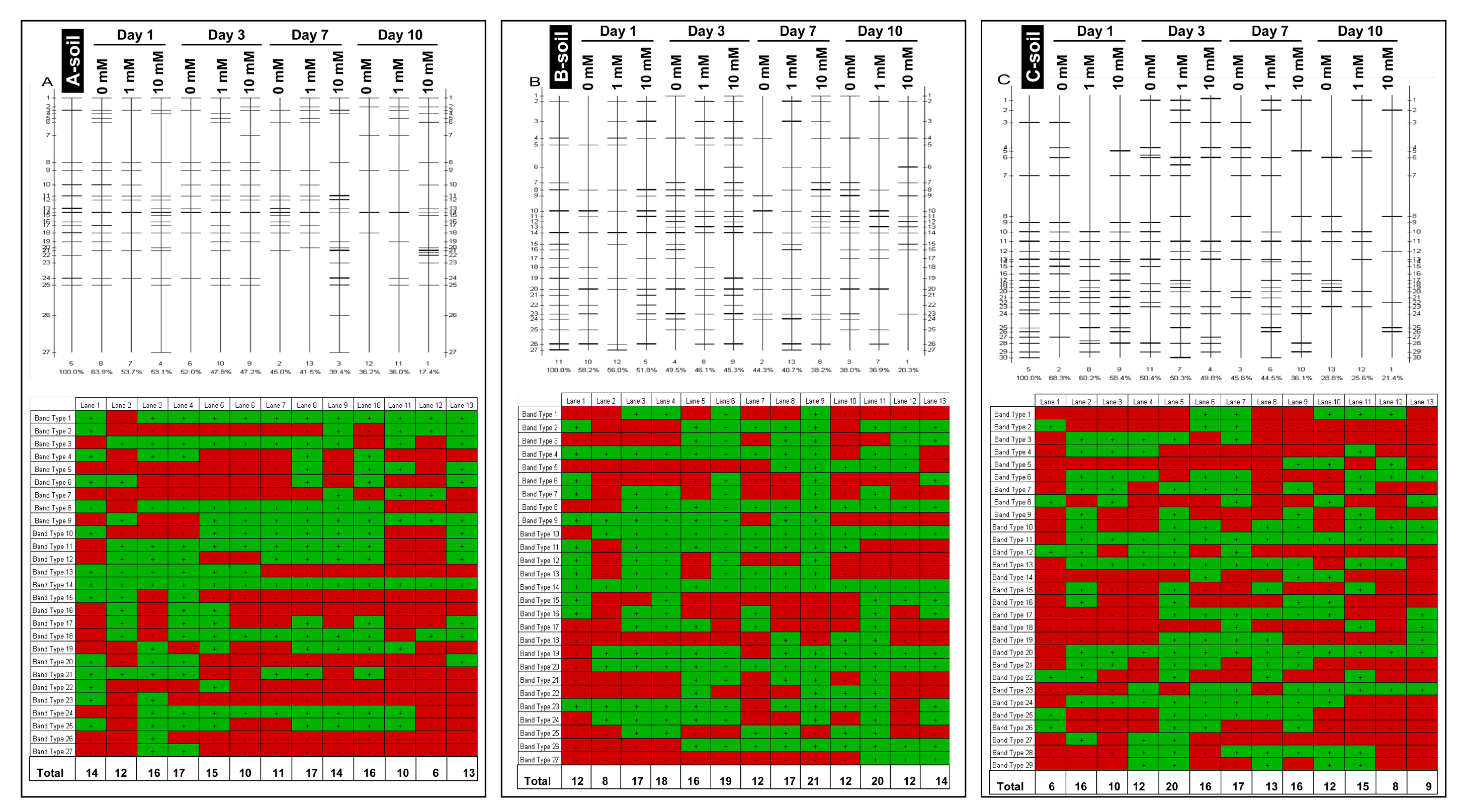

Supplement: Figure S2 — Illustration of the total amplified bands on the DGGE profile shown in Fig. 2. A, B, and C correspond to Figs. 3B, 3C, and 3D, respectively. (TIF) [file pone.0073778.s002.tif]

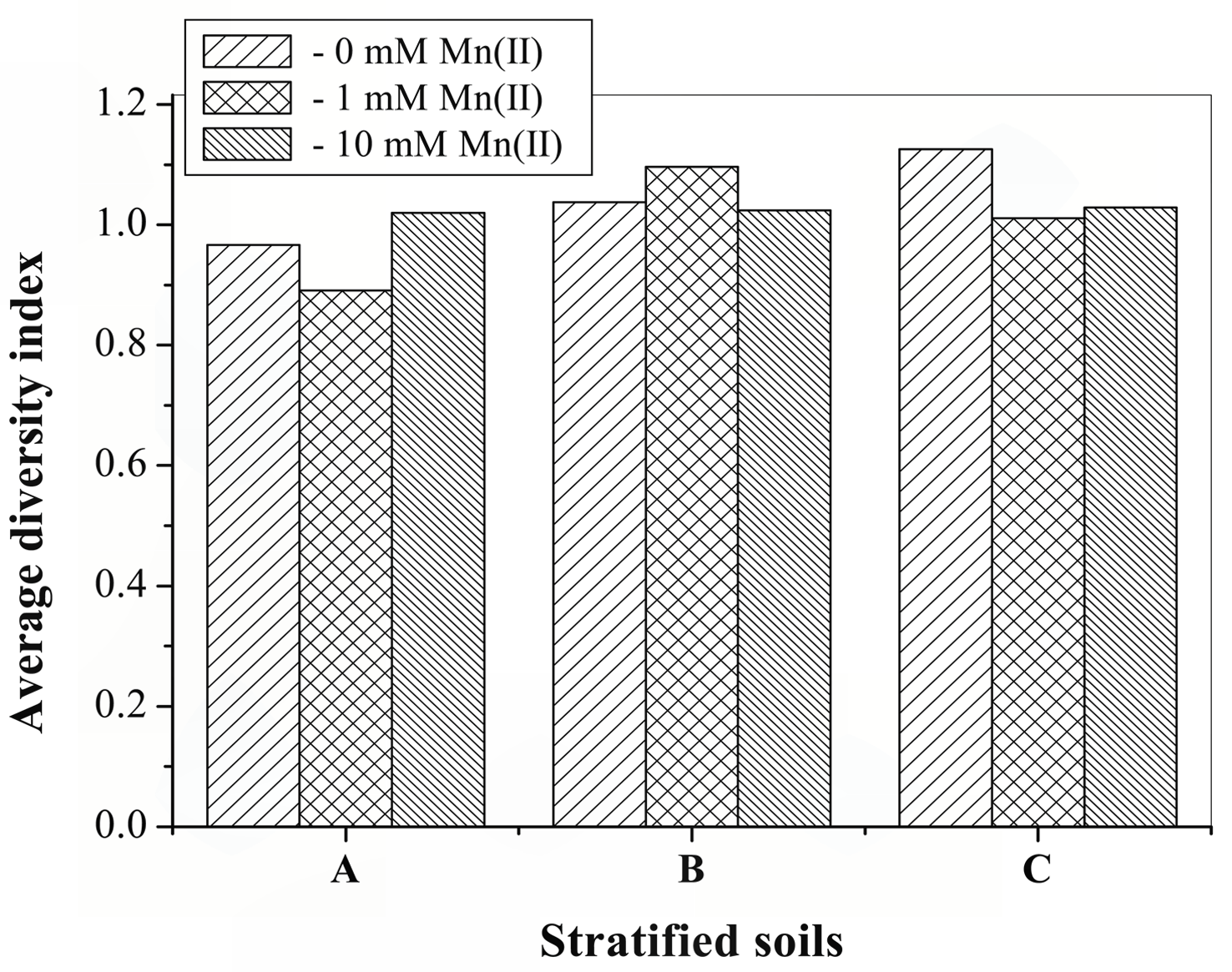

Supplement: Figure S3 — Average diversity index of Mn(II)-enriched soil samples calculated from B, C, and D in Fig. 5. (TIF) [file pone.0073778.s003.tif]

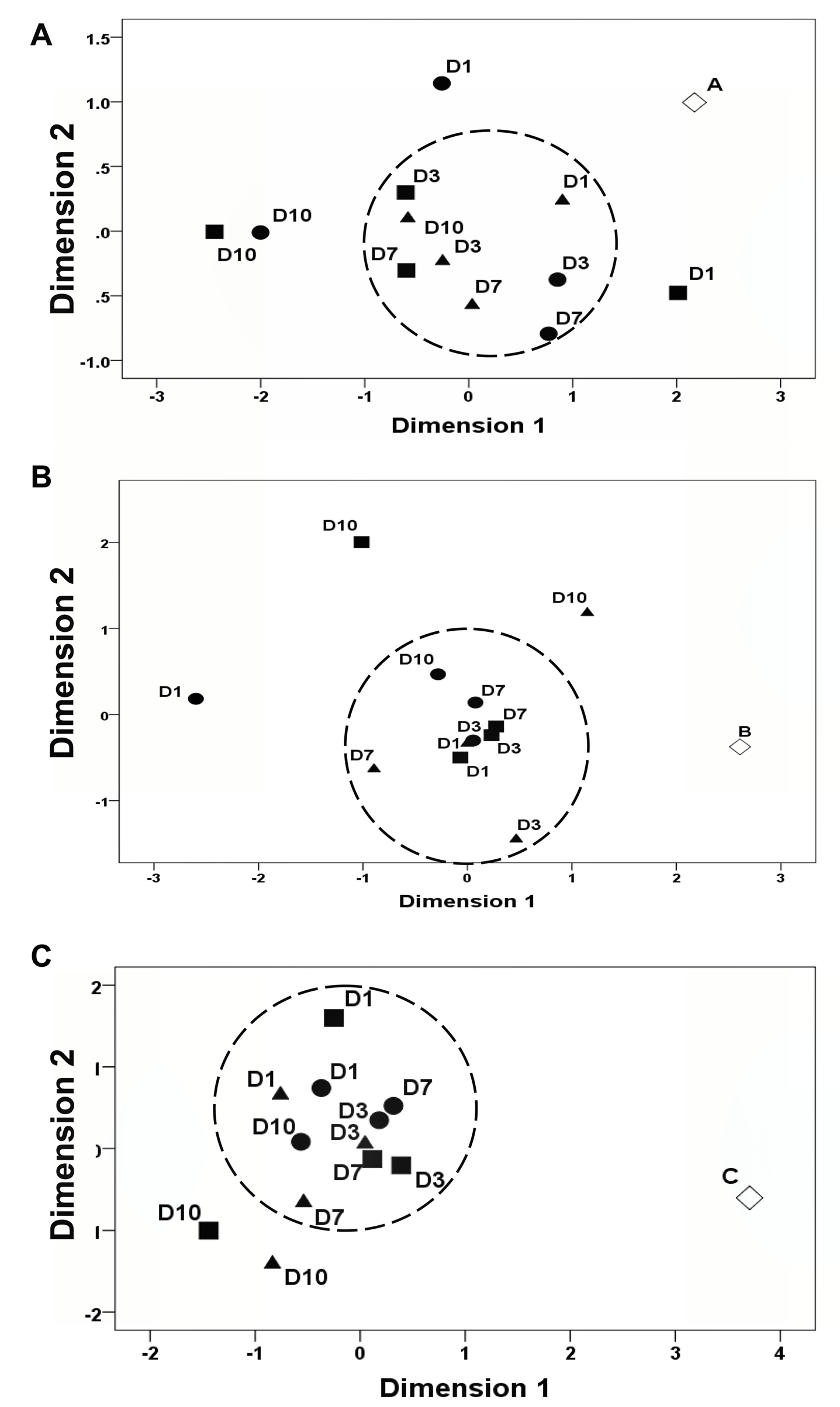

Supplement: Figure S4 — Two-dimensional plots of the MDS analysis results of the DGGE patterns of Mn(II)-enriched soil samples from different depths (represented by A, B, and C) in Figs. 2B, C, and D . ◊, original soil; •, 0 mM Mn(II)-enriched soil; ▪, 1 mM Mn(II)-enriched soil; and ▴, 10 mM Mn(II)-enriched soil. (TIF) [file pone.0073778.s004.tif]

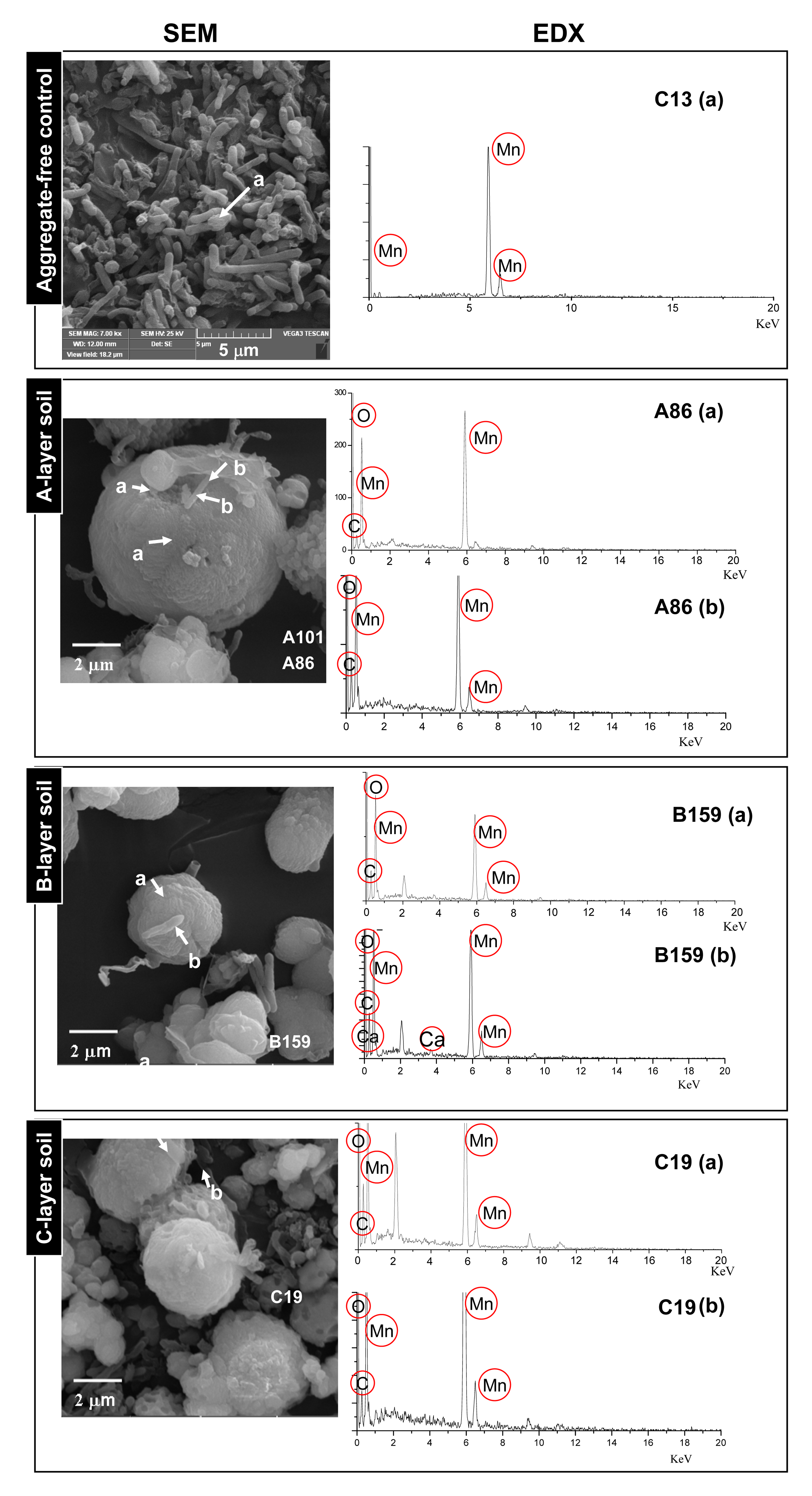

Supplement: Figure S5 — SEM images of the mixture of bacteria and Mn oxides as well as EDX spectra of the corresponding selected areas. A86, B159, and C19 illustrate the formation of Mn oxide aggregates; C13 represents a sample without the formed Mn oxide aggregates. (TIF) [file pone.0073778.s005.tif]
