# Supplementary material for: Population Structure of Manganese-Oxidizing Bacteria in Stratified Soils and Properties of Manganese Oxide Aggregates under Manganese–Complex Medium Enrichment
Source: PLoS One. 2013 Sep 12;8(9):e73778. doi: 10.1371/journal.pone.0073778 (PMC3772008; doi:10.1371/journal.pone.0073778)
Supplement: Table S1 — Summary of the 16S rRNA gene sequences obtained from the predominant bands in the DGGE gel from different soil horizons and the closest match to the NCBI nucleotide sequence database (GenBank)a. (DOC) [file pone.0073778.s006.doc]

**Table S1**. Summary of the 16S rRNA gene sequences obtained from the predominant bands in the DGGE gel from different soil horizons and the closest match to the NCBI Nucleotide Sequence database (GenBank)^a^

| Band number | Closest matched sequences | GenBank accession number | Similarity (%) | Phylogenetic affiliation^b^ |
| --- | --- | --- | --- | --- |
| **A** | | | | |
| A1 | *Enterobacter* sp*.* A12C5 | HQ246256 | 91 | Gammaproteobacteria |
| A2 | Uncultured *Ralstonia* sp*.* clone BF65B_B66 | HM141349 | 76 | Betaproteobacteria |
| A3 | *Bacillus pumilus* strain Hb-0509 | GQ487539 | 84 | Firmicutes |
| A4 | *Bacillus* sp*.* 7A9S1 | HQ246262 | 95 | Firmicutes |
| A5 | *Bacillus* sp*.* JC39 | FN430835 | 83 | Firmicutes |
| A6 | *Escherichia* c*oli* strain G6B153 | GU646192 | 94 | Gammaproteobacteria |
| A7 | Uncultured *Bacillus* sp. isolate | GQ289411 | 83 | Firmicutes |
| A8 | *Erwinia pyrifoliae* strain Ejp617 | AM931292 | 83 | Gammaproteobacteria |
| A9 | *Bacillus* sp*.* BR021 | FJ889572 | 88 | Firmicutes |
| A10 | *Janthinobacterium* sp*.* M169 | HM113654 | 84 | - |
| A11 | *Shigella flexneri* strain RI25 | FJ789704 | 87 | Gammaproteobacteria |
| **B** | | | | |
| B1 | *Sphingobacterium* sp*.* DDT-6 | EU927288 | 97 | Bacteroidetes |
| B2 | Uncultured bacterium clone 4 | FJ904656 | 100 | - |
| B3 | *Escherichia coli* strain G6B153 | GU646192 | 96 | Gammaproteobacteria |
| B4 | Soil bacterium Chlo-S1T-M1LLLSSL-1 | EU515373 | 84 | - |
| B5 | Uncultured bacterium clone 16slp92-01e03 | GQ159514 | 100 | - |
| B6 | Uncultured Gammaproteobacterium clone C4-00yk7 | EU810925 | 85 | Gammaproteobacteria |
| B7 | *Bacillus* sp. ICB415 | HM748070 | 96 | Firmicutes |
| B8 | *Bacillus pichinotyi* strain HPCPS12 | HM072318 | 86 | Firmicutes |
| B9 | Uncultured bacterium clone SEW-C-G19 | EF659133 | 92 | - |
| B10 | Uncultured Proteobacterium clone MS101A1_G09 | EF703370 | 98 | Proteobacterium |
| B11 | Uncultured *Vibrio* sp*.* clone YDB21 | DQ452589 | 94 | Gammaproteobacteria |
| B12 | Uncultured γ-Proteobacterium clone 1HP1-K3 | EU780413 | 96 | Gammaproteobacteria |
| B13 | *Bacillus anthracis* strain RSNPB13 | HM588153 | 90 | Firmicutes |
| **C** | | | | |
| C1 | Uncultured bacterium clone BFV05_599 | GU100706 | 100 | - |
| C2 | Uncultured Enterobacteriales bacterium cloneE10-01QJF | EU434894 | 100 | Gammaproteobacteria |
| C3 | Uncultured Gammaproteobacterium clone C4-00yk7 | EU810925 | 91 | Gammaproteobacteria |
| C4 | *Erwinia chrysanthemi* strain DSM 4610 | AJ233412 | 95 | Gammaproteobacteria |
| C5 | *Klebsiella oxytoca* strain CECRI-IOC43 | HM756496 | 97 | Gammaproteobacteria |
| C6 | *Lysinibacillus* sp*.* INBio2924C | HM771060 | 98 | Firmicutes |
| C7 | *Acinetobacter* sp*.* PRB3 | GQ426948 | 96 | Gammaproteobacteria |
| C8 | *Terribacillus halophilus* strain AK39315 | HQ234337 | 88 | Firmicutes |
| C9 | *Simplicispira* *limi* strain ST3 | FJ982926 | 90 | Betaproteobacteria |
| C10 | *Pseudomonas* sp*.* THt15-7 | HQ290098 | 96 | Gammaproteobacteria |
| C11 | *Pseudomonas putida* strain SKG-1 | HQ259593 | 97 | Gammaproteobacteria |
| C12 | *Escherichia albertii* strain KWB10-151 | HM194885 | 86 | Gammaproteobacteria |
| C13 | *Lysobacter* sp*.* HJHJ-0803 | HM063960 | 90 | Gammaproteobacteria |

^a^ DGGE bands correspond to 37 bands on DGGE profile of Fig. 2.

^b^ “-”: Unclassified group.
